# Lipopolysaccharide induces a fibrotic-like phenotype in endothelial cells

**DOI:** 10.1111/jcmm.12066

**Published:** 2013-05-02

**Authors:** César Echeverría, Ignacio Montorfano, Daniela Sarmiento, Alvaro Becerra, Felipe Nuñez-Villena, Xavier F Figueroa, Claudio Cabello-Verrugio, Alvaro A Elorza, Claudia Riedel, Felipe Simon

**Affiliations:** a Departamento de Ciencias Biológicas, Facultad de Ciencias Biológicas & Facultad de Medicina, Universidad Andres BelloSantiago, Chile; b Millennium Institute on Immunology and ImmunotherapySantiago, Chile; c Departamento de Fisiología, Facultad de Ciencias Biológicas, Pontificia Universidad Católica de ChileSantiago, Chile

**Keywords:** Inflammation, endothelial dysfunction, lipopolysaccharide, fibrosis

## Abstract

Endothelial dysfunction is crucial in endotoxaemia-derived sepsis syndrome pathogenesis. It is well accepted that lipopolysaccharide (LPS) induces endothelial dysfunction through immune system activation. However, LPS can also directly generate actions in endothelial cells (ECs) in the absence of participation by immune cells. Although interactions between LPS and ECs evoke endothelial death, a significant portion of ECs are resistant to LPS challenge. However, the mechanism that confers endothelial resistance to LPS is not known. LPS-resistant ECs exhibit a fibroblast-like morphology, suggesting that these ECs enter a fibrotic programme in response to LPS. Thus, our aim was to investigate whether LPS is able to induce endothelial fibrosis in the absence of immune cells and explore the underlying mechanism. Using primary cultures of ECs and culturing intact blood vessels, we demonstrated that LPS is a crucial factor to induce endothelial fibrosis. We demonstrated that LPS was able and sufficient to promote endothelial fibrosis, in the absence of immune cells through an activin receptor–like kinase 5 (ALK5) activity–dependent mechanism. LPS-challenged ECs showed an up-regulation of both fibroblast-specific protein expression and extracellular matrix proteins secretion, as well as a down-regulation of endothelial markers. These results demonstrate that LPS is a crucial factor in inducing endothelial fibrosis in the absence of immune cells through an ALK5-dependent mechanism. It is noteworthy that LPS-induced endothelial fibrosis perpetuates endothelial dysfunction as a maladaptive process rather than a survival mechanism for protection against LPS. These findings are useful in improving current treatment against endotoxaemia-derived sepsis syndrome and other inflammatory diseases.

## Introduction

Sepsis syndrome, including severe sepsis and septic shock, is the most prevalent cause of mortality in critically ill patients admitted to the intensive care units 1. It is well accepted that the detrimental effects of sepsis syndrome progress as a consequence of an over activation of the immune system, including activation of macrophages and phagocytes, as well as secretion of cytokines and reactive oxygen species (ROS) 1, 2. For that reason, the current treatment for sepsis syndrome includes anti-inflammatory drugs and immune system suppressors. However, the mortality index of sepsis is still high, suggesting that immune system–independent pathways also contribute to sepsis syndrome pathogenesis 3, 4. In line with this phenomenon, impairment of ECs function appears to be an important factor during sepsis syndrome progression 5, 6. In fact, evidence indicates that endothelial dysfunction is a crucial factor in the multi-organic failure observed in sepsis syndrome 7, 8.

Endotoxaemia, which is an important cause of sepsis syndrome, is produced by the deposition of large amounts of the gram-negative bacterial endotoxin LPS 9–12. During endotoxaemia, the LPS on circulating bacteria in the bloodstream inevitably interacts with ECs. LPS recognition by ECs triggers intracellular signalling events including ROS generation and activation of NF-κB-dependent protein synthesis 7, 13, 14.

For more than 20 years, it has been widely accepted that LPS induces endothelial cell death 3, 13, 15, 16. In recent years, we have reported that ECs exposed to LPS undergo cell death by means of an NAD(P)H oxidase–dependent ROS generation mechanism 13, 17. Although moderate ROS generation facilitates normal cellular function, overproduction results in deleterious changes in gene expression and protein malfunction, thus promoting endothelial damage 13, 17–21. Nevertheless, we have observed that, even in the presence of a high concentration of LPS, endothelial death is never complete, with a significant portion of cells remaining alive and fully attached to the plate surface 17. However, the cellular mechanism enabling EC resistance to LPS challenge is not known.

Inflammatory conditions, similar to the environment generated during sepsis syndrome, prompt ECs to adopt fibroblast features through a process known as endothelial-to-mesenchymal transition (EndMT). Although EndMT is typically observed during embryogenesis, in adult organisms, it occurs during pathological processes, such as renal fibrosis, cardiac and pulmonary fibrosis, and cancer 22–26. As a consequence of EndMT, changes in protein synthesis patterns occur. Endothelial markers, such as CD31/PECAM and VE-cadherin, are down-regulated, whereas fibroblast-specific genes, such as α-smooth muscle actin (α-sma) and fibroblast-specific protein 1 (FSP-1), are up-regulated. Furthermore, proteins that constitute the extracellular matrix (ECM), including fibronectin (FN) and type I and III collagen, are increased. In addition, morphological changes have been observed during EndMT, when the endothelial monolayer is disrupted, cell-to-cell contact is lost and ECs acquire a fibroblast-like spindle shape 27–30.

Several studies have demonstrated that transforming growth factor beta 1 (TGFβ1) signalling mediates EndMT 23, 24, 27–30. In addition, recent studies have shown that TGFβ2 signalling can also induce EndMT 27, 31, 32. These findings implicate TGFβ receptor type I [TβRI; activin receptor–like kinase 5 (ALK5)] activation and subsequent smad2/3 proteins phosphorylation 33, 34. Furthermore, others cytokines such as IL-1β and TNF-α seem to perform a synergistic role in EndMT with TGFβ, suggesting a crosstalk between pro-inflammatory mediators generated by immune cells and pro-fibrotic signals 31, 35, 36.

Although ECs exposed to LPS are subjected to an inflammatory environment, we consider here the possibility that LPS exposure, in the absence of immune cells, initiates conversion of ECs into fibroblasts. In fact, ECs resistant to LPS challenge do not look like typical EC, but instead show a spindle-shaped phenotype resembling fibroblasts, suggesting that LPS-treated ECs could enter into an EndMT-like process 17. Therefore, the aim of this study was to investigate the fibrotic effect of LPS on human ECs in the absence of immune cells and explore the underlying mechanism.

Here, we demonstrate that LPS is capable of inducing a fibrotic effect in ECs through an ALK5 activity–dependent mechanism. These findings contribute to our understanding of the mechanisms involved in the endothelial dysfunction observed during sepsis syndrome pathogenesis.

## Materials and methods

Details of all procedures are provided in Additional Supporting Information.

### Cell and vessel culture

#### Primary cell culture

Human umbilical vein endothelial cells (HUVEC) were isolated by collagenase (0.25 mg/ml) digestion from freshly obtained umbilical cord veins from normal pregnancies, after patient's informed consent. The Commission of Bioethics and Biosafety of Universidad Andres Bello approved all experimental protocols. The investigation also conforms with the principles outlined in the Declaration of Helsinki.

#### Blood vessel culture

Intact veins from human umbilical cord were cultured and perfused with medium containing the vehicle or LPS. Vein ends were clamped to avoid solution escape. A detailed protocol is described in Figure S1.

### RNA isolation and quantitative real-time PCR

Quantitative PCR experiments were performed to measure CD31, VE-cadherin, α-sma, FSP-1, fibronectin and type III collagen mRNA levels in HUVEC cells. Total RNA was extracted with Trizol according to the manufacturer's protocol (Invitrogen, Carlsbad, CA, USA). DNAse I-treated RNA was used for reverse transcription using the Super Script II Kit (Invitrogen). Equal amounts of RNA were used as templates in each reaction. QPCR was performed with the SYBR Green PCR Master Mix (AB Applied Biosystems, Foster City, CA, USA). Assays were run using a Rotor-gene system (Corbet Research, Sydney, Australia) instrument. Data are presented as relative mRNA levels of the gene of interest normalized to relative levels of 28S mRNA.

### Western blot procedures

Untreated or LPS-treated ECs were lysed in cold lysis buffer, and then proteins were extracted. Supernatants were collected and stored in the same lysis buffer. Protein extract and supernatant were subjected to sodium dodecyl sulfate polyacrylamide gel electrophoresis and resolved proteins were transferred to a nitrocellulose or polyvinylidene fluoride membrane. The blocked membrane was incubated with the appropriate primary antibody, washed twice, and incubated with a secondary antibody. Bands were revealed using a peroxidase-conjugated IgG antibody. Tubulin was used as a loading control. For detection of the phosphorylated form of smad2 (p-smad2), we used an antibody against p-smad2 (p-ser 465/467) and total smad2 was used as a loading control. For a detailed list of antibodies used, see Table S1.

### Immunocytochemistry and immunohistochemistry

#### Fluorescent immunocytochemistry

Cells were washed twice with phosphate buffer saline (PBS) and fixed. The cells were subsequently washed again and incubated with the first primary antibodies. Then, cells were washed twice and incubated with the first secondary antibodies. Samples were mounted with ProLong Gold antifade mounting medium with the fluorescent dye 4'-6-diamidino-2-phenylindole (DAPI) (Invitrogen).

#### Fluorescent immunohistochemistry

Samples obtained from human umbilical cord vein were fixed and permeabilized. Samples were subsequently washed again and incubated with the first primary antibodies. Then, cells were washed twice and incubated with the first secondary antibodies. For a detailed list of antibodies used, see Table S2.

### Cell viability determination

#### MTT assay

Cell viability was evaluated using the 3-(4,5-dimethylthiazol-2-yl)-2,5-diphenyltetrazolium bromide (MTT) colorimetric assay (Invitrogen, Eugene, OR, USA), in which cell viability was quantified by the amount of MTT reduction. After different treatments were performed, cells were co-incubated with anhydrous MTT 4 hrs and then solubilized with an isopropanol/DMSO solution. The optical density value was measured at 540 nm.

#### Propidium iodide incorporation assay

After treatments, total HUVEC cells were harvested by centrifugation at 800 × *g* for 5 min., and the pellet was suspended in 200 μl PBS. Then, cells were washed once with PBS and stained with propidium iodide (PI, 10 μg/ml) for 20 min. at room temperature in the dark. DNA content was analysed with a flow cytometry system (FACSCanto, BD Biosciences, CA, USA). A minimum of 10,000 cells/sample was analysed. PI intensity analysis was performed with FACSDiva software (BD Biosciences).

### Small interfering RNA against ALK5 and transfection

SiGENOME SMARTpool siRNA (four separated siRNAs per human ALK5 transcript) were purchased from Dharmacon (Dharmacon, Lafayette, CO, USA). The following siRNA were used: human ALK5 (siRNA-ALK5) and non-targeting siRNA (siRNA-CTRL) used as a control. In brief, HUVEC were plated overnight in 6- and 24-well plate and then transfected with 5 nmol/l siRNA using DharmaFECT 4 transfection reagent (Dharmacon) according to the manufacturer's protocols in serum-free medium for 24 hrs. After 24–48 transfection, experiments were performed.

### Reagents

Lipopolysaccharide from *E. coli* was purchased from Sigma-Aldrich (St Louis, MO, USA) (0127:B8). ALK5 inhibitor, SB431542 and specific smad3 inhibitor, SIS3 were purchased from Tocris (Ellisville, MO, USA). Apocynin and NAC were purchased from Sigma-Aldrich. TLR4 inhibitor, CLI-095 was purchased from InvivoGen (San Diego, CA, USA). TGFβ1 and TGFβ2 were purchased from R&D Systems (Minneapolis, MN, USA). Buffers and salts were purchased from Merck Biosciences (Darmstadt, Germany).

### Data analysis

All results are presented as the mean ± S.D. An anova followed by the Bonferroni or Dunn's *post hoc* tests was used and considered significant at *P* < 0.05.

## Results

### Lipopolysaccharide is able to induce endothelial fibrosis

**Fig. 1:**
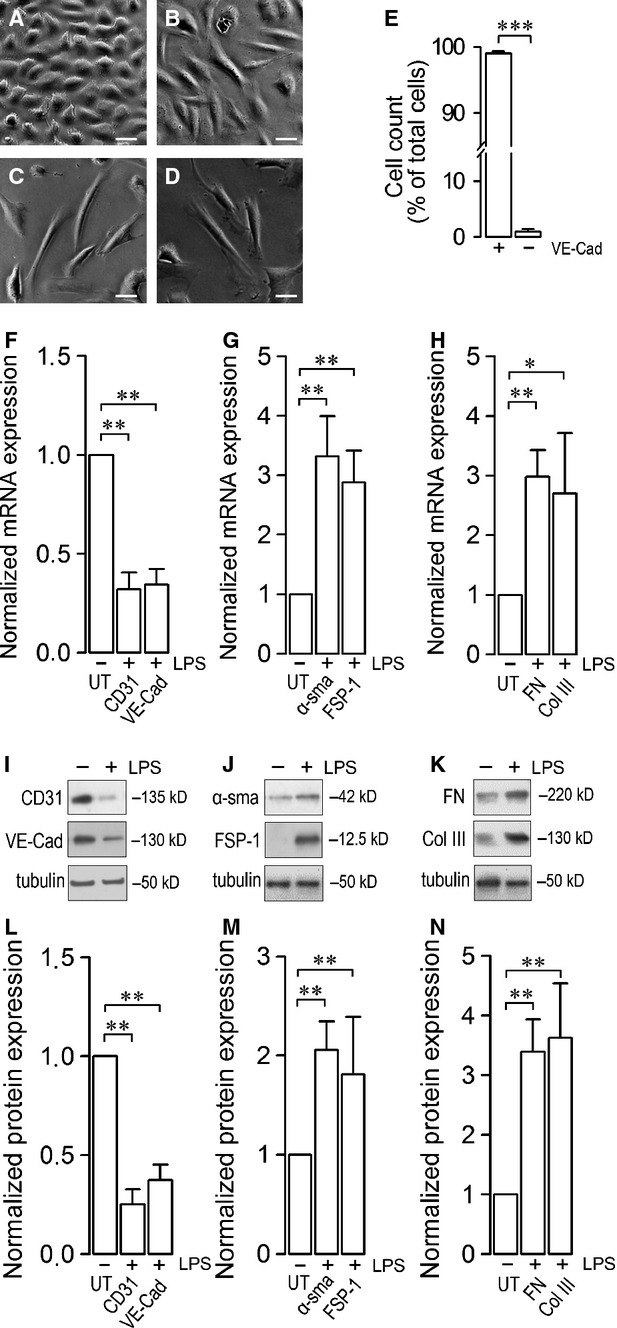
Lipopolysaccharide (LPS)-induced endothelial fibrosis. (**A**–**D**) Morphological changes resembling endothelial-to-mesenchymal transition (EndMT) in human endothelial cells (ECs). Figures show representative phase-contrast images from at least three separates experiments of ECs exposed to vehicle [untreated (UT)] (**A**), 2.5 ng/ml transforming growth factor beta 1 (TGFβ1) (**B**), 10 ng/ml TGFβ2 (**C**) and 20 μg/ml LPS (**D**) for 72 hrs. Bar scale represents 25 μm. (**E**) Primary human umbilical vein endothelial cells (HUVEC) cultures were subjected to immunocytochemistry experiments to identify ECs as VE-Cad-positive cells (VE-Cad^+^) and non-endothelial as VE-Cad-negative cells (VE-Cad^−^). Data are expressed as percentage of total cells counted. Several independent experiments were counted (*N* = 10). Statistical differences were assessed by student's *t*-test (Mann–Whitney). ***P < 0.0001. (**F**–**H**) ECs were exposed to LPS for 72 hrs and mRNA expression of endothelial markers CD31 and VE-cadherin (VE-cad) (**F**), fibrotic markers α-sma and fibroblast-specific protein 1 (FSP-1) (**G**) and fibronectin (FN) and type III collagen (Col III; **H**) were analysed. Determinations were performed at least in triplicates and results are expressed normalized relative to 28S mRNA expression. Statistical differences were assessed by one-way anova (Kruskal–Wallis) followed by Dunn's *post hoc* test. *P < 0.05 and **P < 0.01 against UT condition. Graph bars show the mean ± S.D. (*N* = 3–7). (**I**–**N**) ECs were exposed to LPS for 72 hrs and protein expression was analysed. (**I**–**K**) Representative images from western blot experiments performed for the detection of endothelial markers CD31 and VE-cad (**I**), fibrotic markers α-sma and FSP-1 (**J**) and extracellular matrix (ECM) proteins FN and type III collagen (Col III; **K**). (**L**–**N**) Densitometric analyses of the experiments shown in (**I**–**K**) respectively. Protein levels were normalized against tubulin and data are expressed relative to the UT condition. Statistical differences were assessed by a one-way anova (Kruskal–Wallis) followed by Dunn's *post hoc* test. *P < 0.05 and **P < 0.01 against the UT condition. Graph bars show the mean ± S.D. (*N* = 3–6).

Our first aim was to identify an EndMT-like process in ECs exposed to LPS. Untreated ECs showed a round short-spindle morphology with a cobblestone appearance (Fig. 1A). As TGFβ1 and TGFβ2 are the most studied EndMT inducers, we used both as a positive control. A spindle-shaped phenotype was observed upon both TGFβ1 (Fig. 1B) and TGFβ2 (Fig. 1C) treatment, suggesting that an EndMT process had taken place. In line with these observations, ECs exposed to LPS showed a similar morphological change as that observed in the presence of TGFβ1 and TGFβ2 (Fig. 1D), suggesting that LPS treatment could be triggering a similar process as TGFβ. As reported previously, TGFβ1 treatment exhibited a dose-dependent toxic effect (Figure S2) 37. To ensure that our results are not because of contaminating cells, such as fibroblasts or mesenchymal-like cells, we performed an exhaustive examination of our EC culture enrichment. Using VE-cadherin as a specific endothelial marker, we found that >99% of cells in our EC cultures were VE-cadherin positive, demonstrating that our primary EC cultures are highly enriched in ECs and virtually devoid of any contaminating cells (Fig. 1E).

To test whether LPS treatment changed mRNA levels of genes involved in EndMT, we performed RT-qPCR experiments. LPS-treated ECs showed a decrease in mRNA expression of endothelial markers CD31 and VE-cadherin (Fig. 1F), while the fibrotic markers α-sma and FSP-1 were increased (Fig. 1G). One of the main characteristics of fibrosis is the overproduction of ECM proteins. Therefore, we measured the mRNA expression of the ECM proteins fibronectin and collagen and found that both increased in LPS-treated ECs (Fig. 1H).

Next, we investigated whether LPS treatment could induce EndMT at the protein level. In concordance with the changes in mRNA expression, LPS-treated ECs showed a decrease in the protein level of CD31 and VE-cadherin (Fig. 1I and L). Moreover, LPS challenge induced an increase in the protein level of α-sma and FSP-1 (Fig. 1J and M). Basal expression of α-sma was detected in the untreated condition and was found to be consistent with previously reported levels 38, 39 (Fig. 1G). To analyse the oversecretion of ECM proteins, we measured the fibronectin and collagen protein levels in the supernatant of EC cultures. LPS-treated ECs showed an increase in fibronectin and type III collagen secretion (Fig. 1K–N).

**Fig. 2:**
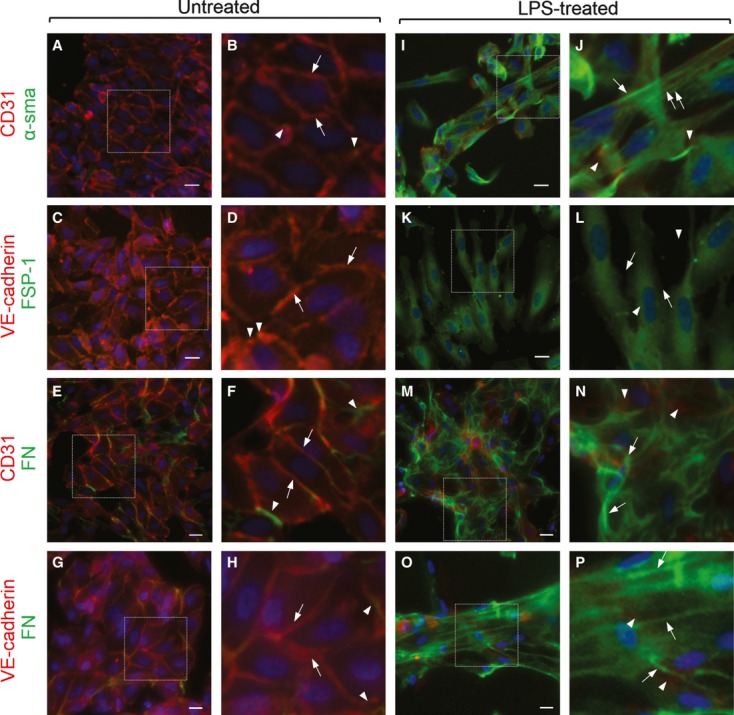
Cellular distribution of proteins involved in lipopolysaccharide (LPS)-induced endothelial fibrosis. Representative images from experiments of untreated (**A**–**H**) or 20 μg/ml LPS-treated (**I**–**P**) endothelial cells (ECs) for 72 hrs. CD31 or VE-cadherin (red), and α-sma, fibroblast-specific protein 1 (FSP-1), or fibronectin (FN; green) were detected. The box depicted in (**A**, **C**, **E** and **G**) indicates the magnification shown in (**B**, **D**, **F** and **H**) respectively. Arrows indicate CD31 (**B** and **F**) or VE-Cadherin (**D** and **H**) labelling at the plasma membrane, whereas arrowheads indicate α-sma (**B**), FSP-1 (**D**) or FN (**F** and **H**) staining, indicating basal expression of fibrotic markers (**B** and **D**) or extracellular matrix (ECM) proteins (**F** and **H**). The box depicted in (**J**, **L**, **N** and **P**) indicates the magnification shown in (**I**, **K**, **M** and **O**) respectively. Arrows indicate α-sma (**J**), FSP-1 (**L**) or FN (**N** and **P**) labelling in plasma membrane, whereas arrowheads indicate CD31 (**J** and **N**) or VE-Cadherin (**L** and **P**) staining from residual endothelial marker expression indicating endothelial-to-mesenchymal transition (EndMT). Nuclei were stained using DAPI. Bar scale represents 10 μm.

To determine the effect of EndMT on the cellular localization and distribution of proteins, we carried out immunocytochemistry experiments. Untreated cells showed CD31 localized principally at the plasma membrane, whereas α-sma was only weakly detected (Fig. 2A and B). VE-cadherin labelling was observed predominantly at the plasma membrane suggesting cell-to-cell association, whereas FSP-1 expression was almost undetectable (Fig. 2C and D). In contrast, LPS-treated ECs showed an increased in α-sma labelling in structures forming stress fibres, which are characteristic of fibrosis, but a decreased in CD31 labelling (Fig. 2I and J). In addition, FSP-1 labelling was severely increased, while VE-cadherin was virtually absent (Fig. 2K and L). Furthermore, we investigated changes in ECM proteins expression upon LPS challenge. As described above, untreated cells showed high CD31 (Fig. 2E and F) and VE-cadherin (Fig. 2G and H) labelling, while fibronectin expression was low (Fig. 2E–H). On the contrary, LPS-treated ECs showed an intense fibronectin labelling with a concomitant decrease in CD31 and VE-cadherin expression (Fig. 2M–P). Unfortunately, it was not possible to study type III collagen by either western blot experiments or immunocytochemistry because our EC cultures are grown in collagen-coated dishes. Similar results were obtained using the well-known EndMT inducer, TGFβ1 (Figure S3), suggesting that both stimuli (LPS and TGFβ) generate analogous changes. Significant staining was not detected when the primary antibody was omitted (Figure S4).

### Lipopolysaccharide-induced endothelial fibrosis in endothelial monolayers from intact blood vessels

**Fig. 3:**
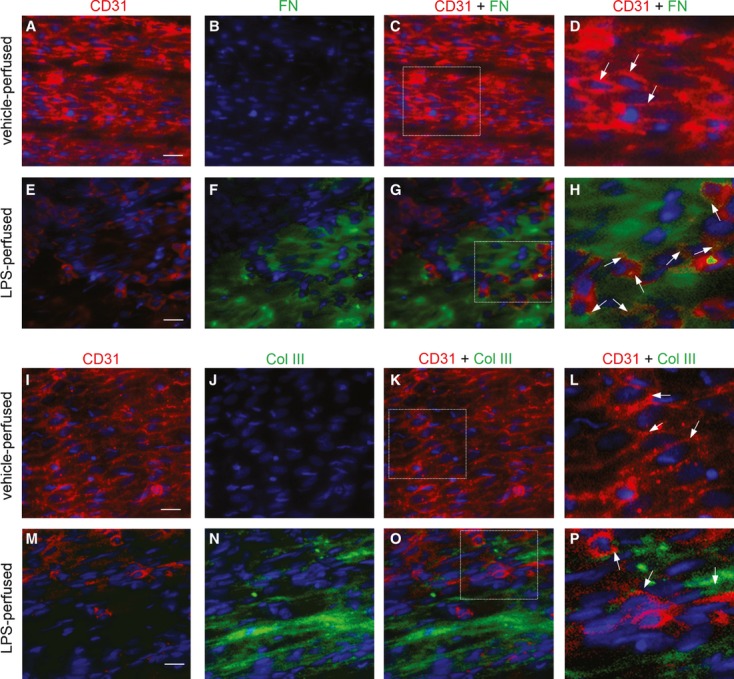
Lipopolysaccharide (LPS)-induced endothelial fibrosis in intact whole blood vessels. (**A**–**P**) endothelial cells (ECs) from intact whole blood vessels perfused with a solution containing vehicle (**A**–**D**) and (**I**–**L**) or 20 μg/ml LPS (**E**–**H**) and (**M**–**P**) for 48 hrs. CD31 (**A**, **E**, **I** and **M**), fibronectin (FN; **B** and **F**) and Col III (**J** and **N**) were detected and the images of CD31 + FN (**C** and **G**) and CD31 + Col III (**K** and **O**) were merged. The box depicted in (**C**, **G**, **K** and **O**) indicates magnification (**D**, **H**, **L** and **P**) respectively. Arrows indicate CD31 labelling at the plasma membrane (**D** and **L**) or colocalization of CD31 and FN (**H**) or CD31 and Col III (**P**) indicating endothelial-to-mesenchymal transition (EndMT). Nuclei were stained using Hoechst. Bar scale represents 50 μm.

Primary cell cultures, as shown here, represent a reliable model for studying human endothelia through various experimental approaches and methods of manipulation. Nonetheless, despite experimental limitations, studying ECs in their native environment, complete with the tridimensional complexity of intact tissues, is required to assess the actual physiological relevance of the process being analysed. For that reason, we carried out experiments in which whole umbilical veins were perfused for 48 hrs with medium containing LPS to mimic endotoxaemia (Figure S1). ECs from vehicle-perfused veins showed a clear CD31 labelling at the cell limits (Fig. 3A, C, D, I, K and L, arrow), but low fibronectin (Fig. 3B–D) and type III collagen (Fig. 3J–L) staining. In contrast, ECs from LPS-perfused veins showed a down-regulation of CD31 staining (Fig. 3E, G, H, M, O and P) compared with vehicle-perfused vessels, whereas fibronectin (Fig. 3F–H) and type III collagen (Fig. 3N–P) labelling was greatly increased, showing colocalization of endothelial and fibrotic markers in some cells (Fig. 3H and P arrow). In addition, we did not observe any significant difference in the distribution of CD31 (Figure S5A–D) or tissue morphology (Figure S5E–F) between the endothelial monolayer from veins perfused for 0 hr *versus* 48 hrs, suggesting that perfusion itself did not promote any alterations in vein tissues. Significant staining was not detected when the primary antibody was omitted (Figure S6), indicating that neither non-specific staining nor autofluorescence of internal elastic lamina or other tissue structures interfered with the immunofluorescent signal. These results indicate that LPS was able to initiate endothelial fibrosis in the endothelial monolayer exposed at the inner wall of intact whole vessels.

### Lipopolysaccharide-induced endothelial fibrosis depends on ALK5 activity

**Fig. 4:**
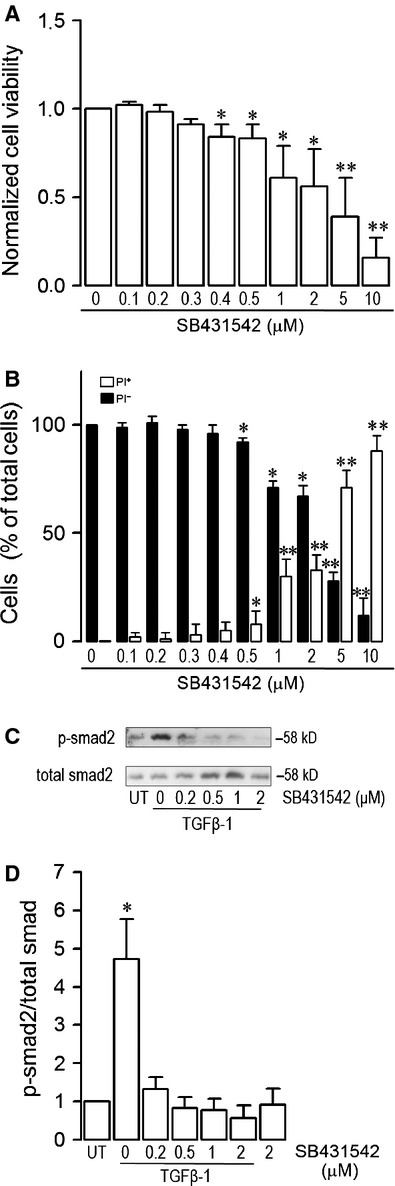
Effects of the activin receptor–like kinase 5 (ALK5) inhibitor, SB431542, on endothelial cells (ECs). Changes in viability of ECs exposed to 0, 0.1, 0.2, 0.3, 0.4, 0.5, 1, 2, 5 and 10 μM ALK5 inhibitor, SB431542 for 72 hrs, evaluated by means of (**A**) MTT assay and (**B**) propidium iodide (PI) incorporation assay. In (**A**), cell viability was expressed relative to the untreated [0 μg/ml transforming growth factor beta 1 (TGFβ1)] condition. In (**B**), cells incorporating PI (empty bars, PI^+^) denote cell death and PI-negative cells (filled bars, PI^+^) denote healthy cells. Statistical differences were assessed by a one-way anova (Kruskal–Wallis) followed by Dunn's *post hoc* test. *P < 0.05 and **P < 0.01 against the untreated (0 μg/ml TGFβ1) condition. Graph bars show the mean ± S.D. (*N* = 3). (**C** and **D**) Dose response of TGFβ1-induced smad2 phosphorylation in the presence of SB431542. Cells were exposed to 5 ng/ml TGFβ1 for 30 min. in the absence or presence of 0, 0.2, 0.5, 1 and 2 μM SB431542 and phosphorylation of smad2 was evaluated. (**C**) Representative images from western blot experiments performed for detection of TGFβ1-stimulated smad2 in the absence or presence of 0, 0.2, 0.5, 1, 2 μM SB431542. (**D**) Densitometric analysis from several experiments as shown in (**C**). Protein levels were normalized against total smad, and the data are expressed relative to the untreated (UT) condition. Statistical differences were assessed by a one-way anova (Kruskal–Wallis) followed by Dunn's *post hoc* test. *P < 0.05 and **P < 0.01 against UT condition. Graph bars show the mean ± S.D. (*N* = 4).

**Fig. 5:**
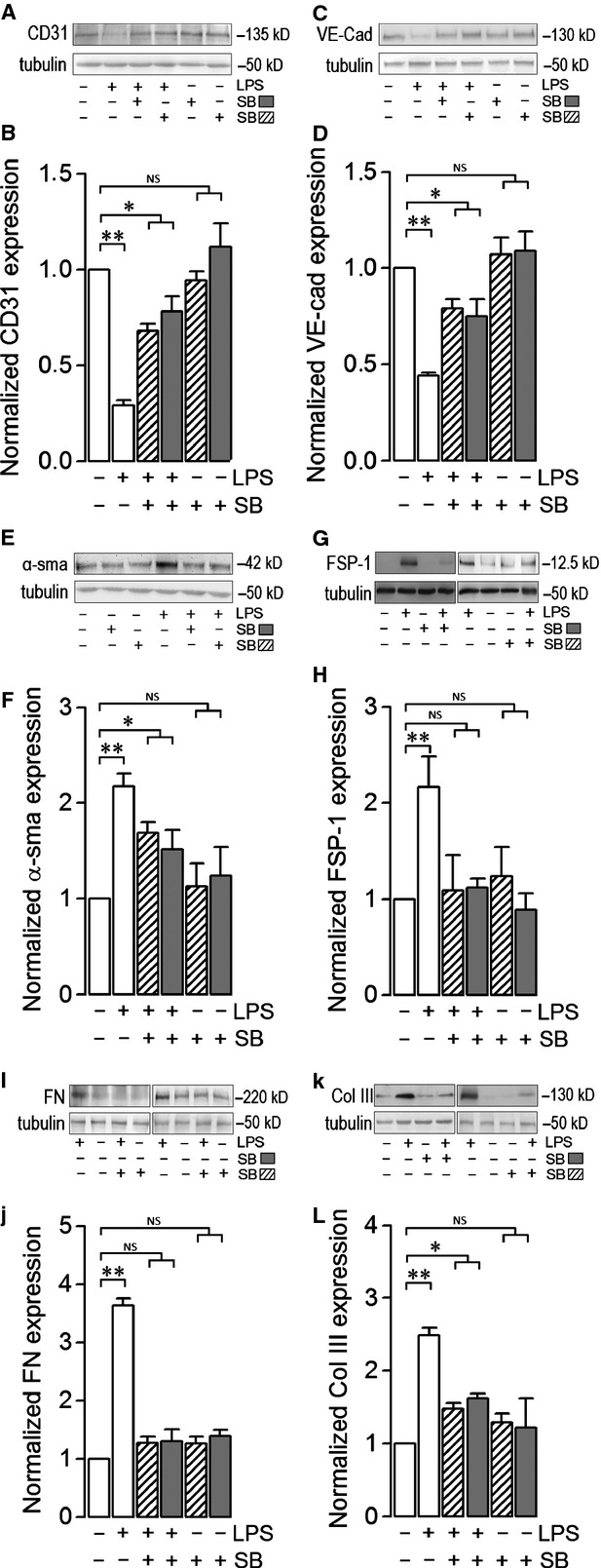
Changes in protein expression during lipopolysaccharide (LPS)-induced endothelial fibrosis are ALK5 activity–dependent. (**A**–**L**) endothelial cells (ECs) were exposed to 20 μg/ml LPS in the absence or presence of the ALK5 inhibitor, SB431542, at 0.2 μM (dashed bars) or 0.5 μM (grey bars) for 72 hrs, and then protein expression was analysed. Representative images from western blot experiments performed for the detection of endothelial markers CD31 (**A**) and VE-cadherin (VE-cad) (**C**), fibrotic markers α-sma (**E**) and fibroblast-specific protein 1 (FSP-1) (**G**) and extracellular matrix (ECM) proteins fibronectin (FN) (**I**) and type III collagen (Col III; **K**). (**B**, **D**, **F**, **H**, **J** and **L**) show densitometric analyses from several experiments, as shown in (**A**, **C**, **E**, **G**, **I** and **K**) respectively. Protein levels were normalized against tubulin, and the data are expressed relative to the untreated (UT) condition. Statistical differences were assessed by a one-way anova (Kruskal–Wallis) followed by Dunn's *post hoc* test. *P < 0.05 and **P < 0.01 against the UT condition. NS: non-significant. Graph bars show the mean ± S.D. (*N* = 3–6).

As previously mentioned, TGFβ1 and TGFβ2 are the most well-studied EndMT inducers. Specifically, TGFβ binds to TβRII, which recruits the TβRI, ALK5. Subsequently, ALK5 phosphorylates smad2 and smad3, which bind smad4 to regulate target gene transcription 33, 34. Thus, our purpose was to investigate whether ALK5 was involved in the mechanism underlying LPS-induced EndMT. To that end, we performed experiments using the ALK5 inhibitor, SB431542. As the concentration of ALK5 inhibitor typically used is toxic to our endothelial cultures (Fig. 4A and B), we determined a lower, non-toxic but still effective concentration of inhibitor to use in our experiments. For that reason, we performed a dose–response curve of SB431542 to evaluate TGFβ1-stimulated smad2 phosphorylation (Fig. 4C and D). Our results demonstrate that lower concentrations (0.2–0.5 μM) of SB431542, which did not have any toxic effects on ECs, were fully effective in abolishing ALK5 kinase activity, as evaluated by TGFβ1-stimulated smad2 phosphorylation state (Fig. 4). In addition, under the lower SB431542 concentrations used in our experimental conditions, basal protein expression in untreated cells was unaffected (Figs 4D and 5, SB431542 alone). Furthermore, the SB431542 concentrations used here ensured a selective ALK5 inhibition rather than ALK4 or ALK7 inhibition 40. The Figure 5 shows ECs exposed to LPS in the absence or presence of ALK5 inhibitor. As we expected, ECs cotreated with LPS and ALK5 inhibitor exhibited a partial but significant abrogation of changes in protein expression compared with LPS-treated cells in the absence of inhibitor. Treatment with the ALK5 inhibitor reduced both LPS-induced down-regulation of CD31 and VE-cadherin (Fig. 5A–D respectively) and LPS-induced up-regulation of α-sma and FSP-1 (Fig. 5E–H respectively). In addition, the increase in the ECM proteins fibronectin and type III collagen was prevented in cells that were co-incubated with LPS and SB431542 (Fig. 5I–L respectively). These results suggest that ALK5 is involved in the LPS-induced EndMT.

**Fig. 6:**
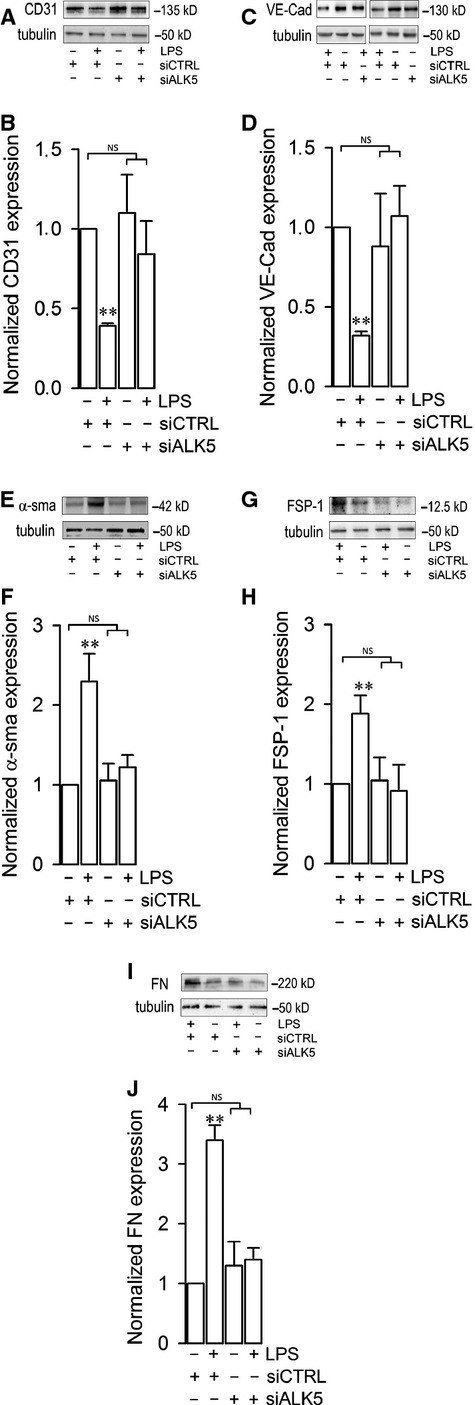
Changes in protein expression during lipopolysaccharide (LPS)-induced endothelial fibrosis are inhibited by transfection with a siRNA against activin receptor–like kinase 5 (ALK5). (**A**–**L**) Endothelial cells (ECs) were exposed to 20 μg/ml LPS and transfected with a siRNA against ALK5 (siALK5) or a non-targeting siRNA (siCTRL) for 72 hrs, and then protein expression was analysed. Representative images from western blot experiments performed for the detection of endothelial markers CD31 (**A**) and VE-cadherin (VE-cad; **C**), fibrotic markers α-sma (**E**) and fibroblast specific protein 1 (FSP-1) (**G**) and extracellular matrix (ECM) proteins fibronectin (FN; **I**). (**B**, **D**, **F**, **H** and **J**) show densitometric analyses from several experiments, as shown in (**A**, **C**, **E**, **G** and **I** respectively). Protein levels were normalized against tubulin, and the data are expressed relative to the untreated (0 μg/ml LPS + siCTRL) condition. Statistical differences were assessed by a one-way anova (Kruskal–Wallis) followed by Dunn's *post hoc* test. *P < 0.05 and **P < 0.01 against the untreated (0 μg/ml LPS + siCTRL) condition. NS: non-significant. Graph bars show the mean ± S.D. (*N* = 3–4).

Despite the evidence obtained using the pharmacological inhibitor SB431542, the use of a molecular biology strategy for ALK5 expression knockdown was necessary to prove unequivocally the participation of the kinase. Thus, to demonstrate the participation of ALK5 in LPS-induced EndMT, cells were transfected with a specific small interference RNA (siRNA) against the human isoform of ALK5 (siALK). The siRNA efficiency in ALK5 expression knockdown was >80% (Figure S7). LPS-treated ECs transfected with siALK5 did not show any decrease in the protein level of CD31 and VE-cadherin (Fig. 6A–D respectively). Moreover, siRNA transfection prevented the increase in the protein level of α-sma and FSP-1 (Fig. 6E–H, respectively), and the ECM protein fibronectin (Fig. 6I and J). As we expected, LPS-treated ECs, transfected with a non-targeting sequence siRNA used as a control (siCTRL), showed similar results to those observed in wild-type cells (Fig. 6). These results corroborate that ALK5 is involved in the LPS-induced EndMT. ALK5 down-regulation >95% for over 72 hrs provoked a toxic effect showing a spindle-shaped appearance (data not shown). Further studies are needed to investigate that issue.

**Fig. 7:**
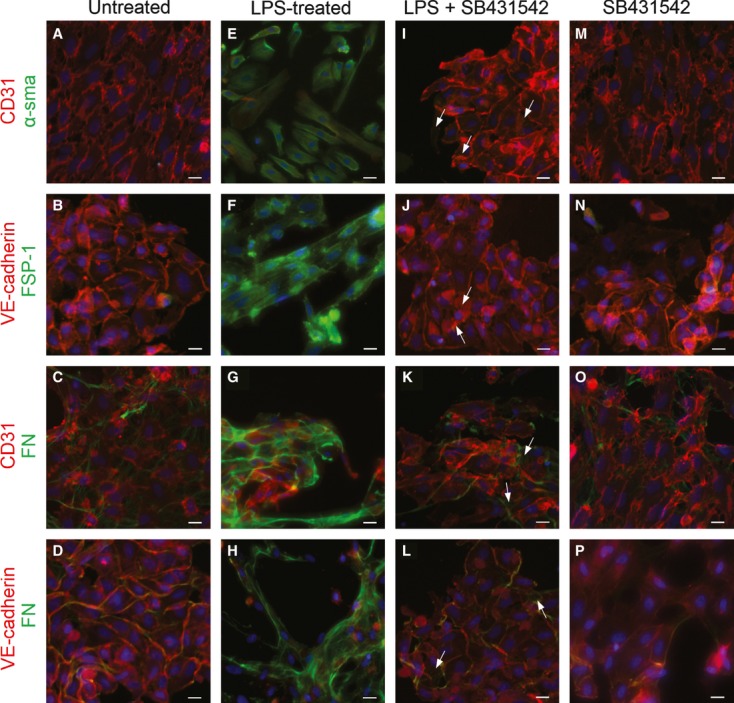
Cellular distribution of proteins involved in lipopolysaccharide (LPS)-induced endothelial fibrosis is activin receptor–like kinase 5 (ALK5) activity–dependent. (**A**–**P**) Representative images from endothelial cells (ECs) untreated (**A**–**D**), LPS-treated (**E**–**H**), LPS- plus SB431542-treated (**I**–**L**) or SB431542-treated alone (**M**–**P**) for 72 hrs. CD31 or VE-cadherin (red), and α-sma, FSP-1, or fibronectin (FN; green) were detected. In LPS- and SB431542-treated ECs, arrows indicate α-sma (**I**), FSP-1 (**J**) or FN (**K** and **L**) staining for fibrotic markers or extracellular matrix (ECM) proteins expression suggesting partial prevention endothelial-to-mesenchymal transition (EndMT). Nuclei were stained using DAPI. Bar scale represents 10 μm.

We performed immunocytochemistry experiments, similar to those shown in Figure 2, to evaluate the effect of ALK5 inhibition on protein distribution in LPS-treated ECs. Our results demonstrate that the ALK5 inhibitor significantly blocks the down- and up-regulation of endothelial and fibrotic markers, respectively, in LPS-treated ECs (Fig. 7). In concordance with the data shown in Figure 2, untreated ECs exhibit CD31 (Fig. 7A and C) and VE-cadherin (Fig. 7B and D) labelling predominantly at the plasma membrane, whereas LPS-treated cells show a strong signal for α-sma, FSP-1 and FN (Fig. 7E–H respectively). Notably, ECs challenged with LPS in the presence of SB431542 did not show an increase in fibrotic marker expression, nor were endothelial markers down-regulated. Endothelial morphology was maintained in the presence of the inhibitor (Fig. 7I–L). The ALK5 inhibitor alone did not affect cell shape or protein marker expression in untreated cells (Fig. 7M–P). All together, these results suggest that LPS-induced EndMT depends on ALK5 activity.

**Fig. 8:**
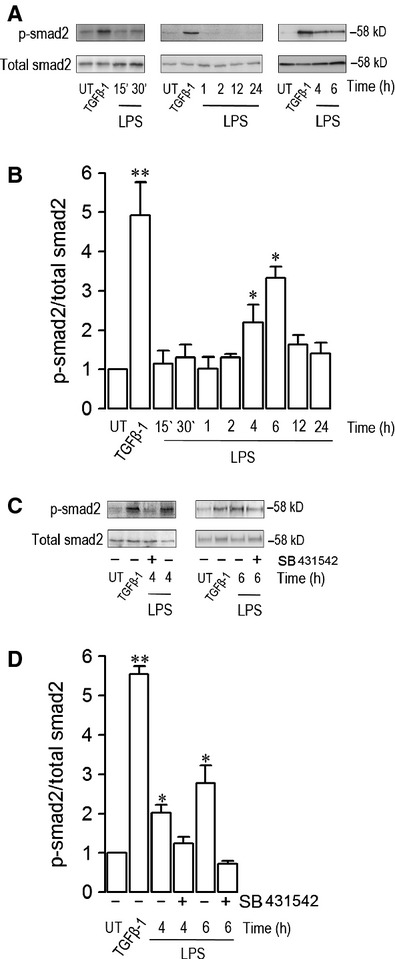
Activin receptor–like kinase 5 (ALK5)–dependent lipopolysaccharide (LPS)-induced smad2 phosphorylation in human endothelial cells (ECs). (**A**–**B**) ECs were exposed to 20 μg/ml LPS, and then smad2 phosphorylation was evaluated. (A) Representative images from western blot experiments performed for detection of smad2 and total smad in untreated, transforming growth factor beta 1 (TGFβ1)-stimulated (30 min.), and 20 μg/ml LPS-treated (15 and 30 min. and 1, 2, 4, 6, 12, 24 hrs) ECs. (**B**) Densitometric analyses from several experiments as shown in (**A**). (**C**–**D**) ECs were exposed to 20 μg/ml LPS in the presence or absence of the ALK5 inhibitor, SB432542, and then smad2 phosphorylation was evaluated. (C) Representative images from western blot experiments performed for the detection of smad2 and total smad in untreated, TGFβ1-stimulated (30 min.), and 20 μg/ml LPS-treated (4 and 6 hrs) ECs in the presence (+) or absence (−) of SB432542. (**B**) Densitometric analyses from several experiments as shown in (**D**). Protein levels were normalized against total smad, and the data are expressed relative to untreated (UT) condition. Statistical differences were assessed by one-way anova (Kruskal–Wallis) followed by Dunn's *post hoc* test. *P < 0.05 and **P < 0.01 against the UT condition. Graph bars show the mean ± S.D. (*N* = 3–4).

As ALK5 kinase activation implies smad phosphorylation, we tested whether LPS was able to induce smad2 phosphorylation. LPS-treated ECs showed smad2 phosphorylation at 4 and 6 hrs after LPS exposure (Fig. 8A and B). In addition, the increase in LPS-induced smad2 phosphorylation was not detected at 12 or 24 hrs (Fig. 8A and B). Interestingly, LPS-induced smad2 phosphorylation was abolished upon addition of the ALK5 inhibitor (Fig. 8C and D), suggesting that LPS-induced smad2 phosphorylation is dependent on ALK5 activity.

**Fig. 9:**
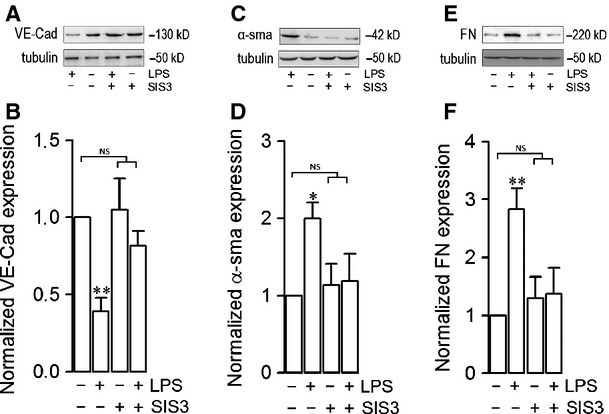
Changes in protein expression during lipopolysaccharide (LPS)-induced endothelial fibrosis are inhibited by the specific inhibitor of smad 3, SIS3. (**A**–**F**) endothelial cells (ECs) were exposed to 20 μg/ml LPS in the absence (−) or presence (+) of the SIS3 for 72 hrs, and then protein expression was analysed. Representative images from western blot experiments performed for the detection of endothelial marker CD31 (**A**), fibrotic marker α-sma (**C**) and extracellular matrix (ECM) protein fibronectin (FN; **E**). **B**, **D** and **F** show densitometric analyses from several experiments, as shown in **A**, **C** and **E** respectively. Protein levels were normalized against tubulin, and the data are expressed relative to the untreated (0 μg/ml LPS) condition. Statistical differences were assessed by a one-way anova (Kruskal–Wallis) followed by Dunn's *post hoc* test. *P < 0.05 and **P < 0.01 against the untreated (0 μg/ml LPS) condition. NS: non-significant. Graph bars show the mean ± S.D. (*N* = 3–5).

Considering that smad3 phosphorylation is linked to fibrotic processes, we addressed the question of whether smad3 activation is involved in the LPS-induced EndMT. For that purpose, we used the specific inhibitor of smad3, SIS3. Our data showed that LPS-treated ECs in the presence of SIS3 failed to decrease the levels of the endothelial marker VE-Cadherin (Fig. 9A and B), and the fibrotic proteins α-sma (Fig. 9C and D) and fibronectin (Fig. 9E and F).

### Lipopolysaccharide-induced endothelial fibrosis depends on TLR4 activation

**Fig. 10:**
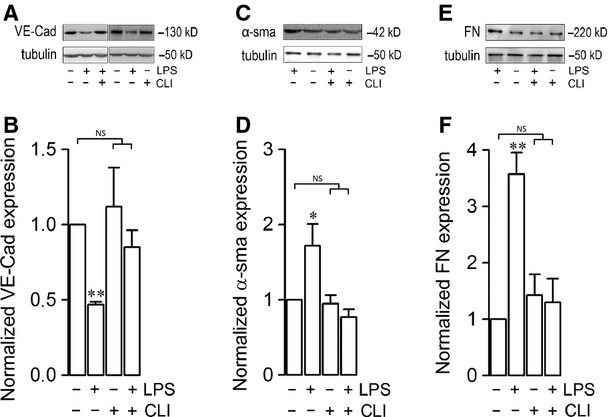
Changes in protein expression during lipopolysaccharide (LPS)-induced endothelial fibrosis are dependent of TLR4 activity. (**A**–**F**) Endothelial cells (ECs) were exposed to 20 μg/ml LPS in the absence (−) or presence (+) of the specific inhibitor of TLR4, CLI-095, for 72 hrs, and then protein expression was analysed. Representative images from western blot experiments performed for detection of endothelial marker CD31 (**A**), fibrotic marker α-sma (**C**), and extracellular matrix (ECM) protein fibronectin (FN; **E**). **B**, **D** and **F** show densitometric analyses from several experiments, as shown in **A**, **C** and **E** respectively. Protein levels were normalized against tubulin, and the data are expressed relative to the untreated (0 μg/ml LPS) condition. Statistical differences were assessed by a one-way anova (Kruskal–Wallis) followed by Dunn's *post hoc* test. *P < 0.05 and **P < 0.01 against the untreated (0 μg/ml LPS) condition. NS: non-significant. Graph bars show the mean ± S.D. (*N* = 3–5).

As the Toll-like receptor 4 (TLR4) is the receptor for LPS, we assessed whether TLR4 is involved in the LPS-induced EndMT. To test this, we used the specific TLR4 inhibitor, CLI-095 (CLI). LPS-treated ECs in the presence of CLI-095 did not show any decrease in the endothelial marker, VE-cadherin (Fig. 10A and B). Moreover, CLI-095 incubation inhibited the increase in the protein level of the fibrotic marker, α-sma (Fig. 10C and D), as well as the ECM protein fibronectin (Fig. 10E and F).

### Lipopolysaccharide-induced endothelial fibrosis depends on NAD(P)H oxidase–dependent ROS production

**Fig. 11:**
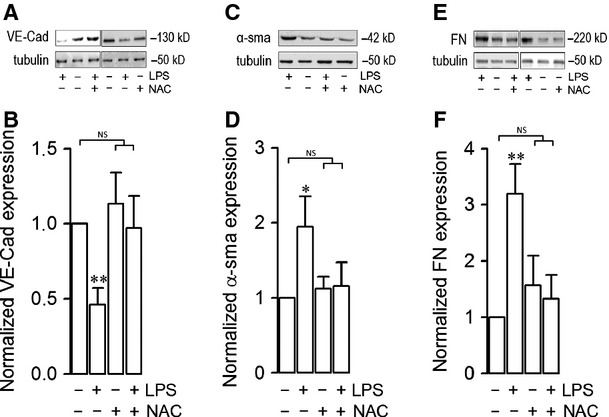
Changes in protein expression during lipopolysaccharide (LPS)-induced endothelial fibrosis are dependent of ROS production. (**A**–**F**) Endothelial cells (ECs) were exposed to 20 μg/ml LPS in the absence (−) or presence (+) of the antioxidant NAC, for 72 hrs, and then protein expression was analysed. Representative images from western blot experiments performed for the detection of endothelial marker CD31 (**A**), fibrotic marker α-sma (**C**) and extracellular matrix (ECM) protein fibronectin (FN; **E**). **B**, **D** and **F** show densitometric analyses from several experiments, as shown in **A**, **C** and **E** respectively. Protein levels were normalized against tubulin, and the data are expressed relative to the untreated (0 μg/ml LPS) condition. Statistical differences were assessed by a one-way anova (Kruskal–Wallis) followed by Dunn's *post hoc* test. *P < 0.05 and **P < 0.01 against the untreated (0 μg/ml LPS) condition. NS: non-significant. Graph bars show the mean ± S.D. (*N* = 3–5).

Considering that ECs exposed to LPS exhibited a significant increase in NAD(P)H oxidase–dependent ROS production 13, we tested whether LPS-induced EndMT is dependent on ROS levels. As shown in Figure 11A–F, LPS-treated ECs in the presence of the antioxidant N-acetyl cysteine (NAC) failed to decrease the levels of the endothelial marker VE-Cadherin (Fig. 11A and B), and the fibrotic proteins α-sma (Fig. 11C and D) and fibronectin (Fig. 11E and F).

**Fig. 12:**
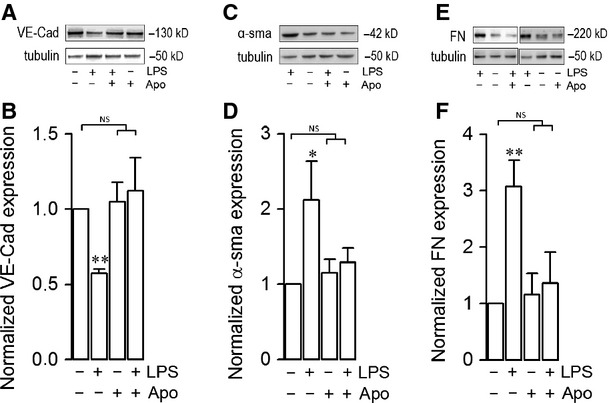
Changes in protein expression during lipopolysaccharide (LPS)-induced endothelial fibrosis are dependent of NAD(P)H oxidase activity. (**A**–**F**) Endothelial cells (ECs) were exposed to 20 μg/ml LPS in the absence (−) or presence (+) of the NAD(P)H oxidase inhibitor, Apocynin (Apo), for 72 hrs, and then protein expression was analysed. Representative images from western blot experiments performed for detection of endothelial marker CD31 (**A**), fibrotic marker α-sma (**C**), and extracellular matrix (ECM) protein fibronectin (FN; **E**). **B**, **D** and **F** show densitometric analyses from several experiments, as shown in **A**, **C** and **E** respectively. Protein levels were normalized against tubulin, and the data are expressed relative to the untreated (0 μg/ml LPS) condition. Statistical differences were assessed by a one-way anova (Kruskal–Wallis) followed by Dunn's *post hoc* test. *P < 0.05 and **P < 0.01 against the untreated (0 μg/ml LPS) condition. NS: non-significant. Graph bars show the mean ± S.D. (*N* = 3–5).

The question whether LPS-induced EndMT requires the activation of NAD(P)H oxidase was examined next. To that end, ECs were incubated with Apocynin (Apo), a compound that specifically blocks the NAD(P)H oxidase activation. LPS-treated ECs in the presence of Apo did not show any decrease in VE-cadherin (Fig. 12A and B). Moreover, incubation with Apo inhibited the increase in the level of α-sma (Fig. 12C and D), and fibronectin (Fig. 12E and F).

These results suggest that LPS-induced EndMT depends on NAD(P)H oxidase–dependent ROS production.

## Discussion

Endothelial dysfunction is an important feature of sepsis syndrome as well as more severe inflammatory pathologies. Thus, understanding the underlying cellular and molecular mechanism of these dysfunctions is crucial to improving the current treatment against sepsis syndrome.

We previously demonstrated that LPS-treated ECs could undergo extensive cell death. In addition, we observed that a significant portion of ECs were resistant to LPS challenge 13, 17. Here, we focused our studies on a population of ECs resistant to LPS exposure and the cellular consequences of their resistance.

In this study, we demonstrated that LPS is able and sufficient to promote endothelial fibrosis in the absence of immune cells, *via* an endothelial-to-mesenchymal transition–like process that is dependent on ALK5 activity. Our results show that LPS can induce up-regulation of fibroblast-specific proteins, as well as down-regulation of endothelial markers. In addition, LPS-challenged ECs strongly up-regulate ECM proteins expression. Furthermore, we demonstrated that LPS-induced endothelial fibrosis requires ALK5 activity. Finally, we demonstrated that LPS is able to induce smad2 phosphorylation.

Systemic inflammation during sepsis syndrome occurs as a result of over activation of the immune system. During bacteraemia, it is widely accepted that LPS promotes its effects indirectly through immune cell such as phagocytes, neutrophils and polymorphonuclears. As ECs express the LPS receptor, TLR4, direct signalling by the endotoxin is plausible. In fact, a number of studies have demonstrated that LPS exerts direct effects on the endothelium, resulting primarily in cell death 3, 13, 15, 16. Along this line, our study demonstrates that LPS is able to modify the protein expression in ECs from a healthy pattern into a pathogenic one in the absence of immune cells. This finding could explain the inefficiency of treatments against bacteraemia based on dampening the immune system.

Sepsis syndrome–induced systemic inflammation results in progressive organ failure, which often culminates in patient fatality 3, 4. In this context, endothelial dysfunction represents, at least in part, a suitable explanation of organ function impairment during sepsis syndrome. However, the mechanism of endothelial dysfunction occurs is far from being understood. Sepsis pathogenesis may partially be explained by the shift in ECs into activated fibroblasts, a process during which the endothelium adopts an aberrant behaviour rather than a loss of function by cell death. Although endothelial fibrosis and EC death occur simultaneously, changes in endothelial function could be more pathogenic than loss of function.

For several years, it was generally accepted that tissue fibrosis occurs as a consequence of fibroblast activation in resident or migrating fibroblasts 41. However, as a consequence of the discovery of epithelial-to-mesenchymal and EndMT, the possibility that the generation of activated fibroblast from differentiated tissues was considered 23, 42–44. Hence, LPS-induced endothelial fibrosis through EndMT emerges as a mechanism to generate activated fibroblasts even in the absence of pre-existing tissue fibroblasts.

Although LPS-induced EndMT could emerge in endothelial cells from all types of blood vessels, considering both the slow blood flow and the blood volume, veins and capillaries appear as the most suitable vessels to be transdifferentiated by LPS exposure. Nonetheless, the mechanism underlying LPS-induced EndMT in veins and capillaries could be different. The modification in capillaries could be of major relevance because alterations in those vessels affect organ irrigation directly. Further experiments are needed to elucidate whether all blood vessels are affected in the same way by the LPS to develop fibrosis.

Normal cells produce and secrete ECM proteins in equilibrium with their degradation 45, 46. During fibrosis, activated fibroblasts secrete high amounts of ECM proteins that overwhelm the cellular capacity for ECM degradation 45, 46. Such imbalance generates morphological changes, differential protein expression patterns and loss of cellular function, among other defects. Thus, an increase in ECM proteins represents evidence for a fibrogenic event. Healthy ECs secrete type IV collagen and low amounts of fibronectin, whereas type I and type III collagen are virtually absent, appearing only after fibrosis has been established 45–48. Here, we demonstrated that LPS-treated ECs overexpress the ECM proteins fibronectin and type III collagen compared with untreated cells, suggesting that LPS promotes ECM overload in ECs, generating changes in endothelial morphology. Further studies are needed to investigate whether LPS-induced endothelial fibrosis impairs specific endothelial functions such as endothelial monolayer permeability, hormone production and NO secretion among others. On the other hand, as ECM proteins are molecular bridges by Gram-negative bacteria to attach to host's surfaces, the LPS-induced ECM overexpression could represent an additional mechanism to contribute to the adherence of bacterial pathogens to fibroblast further enhancing the inflammatory response. Thus, bacterial infection episodes could be more aggressive and difficult to eradicate. Certainly, further studies are needed to test this idea.

A relevant issue is the occurrence of the LPS-induced EndMT in whole organisms during sepsis syndrome. To date, there is no evidence demonstrating endothelial fibrosis during sepsis using *in vivo* models. In animal models of sepsis, renal fibrosis has been observed, but it is difficult to ensure that the renal fibrotic tissue is derived from endothelial cells 4, 49, 50. In addition, the incidence of renal epithelial–mesenchymal transition 44, 51, 52 as well as renal EndMT 53, 54 has been observed in *in vitro* and *in vivo* models of inflammation. However, no results are provided in sepsis-derived inflammatory models. Thus, further studies are needed to demonstrate in *in vivo* models that LPS induces endothelial fibrosis during sepsis syndrome.

Although ALK5 participation in fibrotic processes has been previously demonstrated, mechanisms describing how ALK5 induces fibrosis are not well understood. The results of our study suggest that ALK5 may be activated by LPS-induced TGFβ secretion through an autocrine/paracrine manner and further activate signalling pathways such as smad phosphorylation 55. Concurrently, ALK5 may also be transactivated by other receptors located in the plasma membrane 56. Further studies must be performed to establish the mechanism by which ALK5 participates in LPS-induced EndMT.

An important feature of LPS-induced inflammatory states is ROS generation 13, 17. This process is mediated by modifications in proteins such as kinases, phosphatases, ion channels, transcriptional factors and receptors 20, 21, 57–59. Thus, LPS-induced endothelial fibrosis could be mediated by inflammation-induced ROS production suggesting that antioxidants and reducing agents could be useful for sepsis therapy.

Endothelial fibrosis could not only contribute to the development of sepsis syndrome but also facilitate angiogenic tumour growth. The observation that EndMT can occur in tumour tissues 23, 24, 60, combined with the evidence shown here, suggests the possibility that an inflammatory environment is able to generate carcinogenic progression through an endothelial fibrotic process. Future studies are needed to test this hypothesis.

Taken together, the findings presented here provide evidence that LPS is a sufficient and crucial factor in inducing endothelial fibrosis to generate a reprogramming of endothelial cell protein expression, converting them from a healthy state into a pathogenic one. Understanding the molecular pathways that govern this pathological process will be useful in improving the current treatment against endotoxaemia-derived sepsis syndrome and more severe inflammatory diseases.

## Supporting information

**Table S1.** Primary and secondary antibodies used in western blot experiments.

**Table S2**. Primary and secondary antibodies used in immunocytochemistry and immunohistochemistry.

**Figure S1** Culturing of intact whole blood vessel with differential perfusion. (**A**) Veins from human umbilical cord were isolated and incubated for 48 hrs. External solution contained isotonic medium containing FBS and growth factors. Internal solution was HUVEC medium containing vehicle or 20 μg/ml LPS. External and internal solution were not mixed during the experiments. Solutions were changed frequently. (**B**) Cultured blood vessel was dissected to extract a small portion of vessel wall to exposed ECs monolayers. Samples extracted were 150 mm^2^ approximately. Vessel wall structure was not altered. (**C**) Monolayer samples were placed in a coverslip and immunohistochemistry experiments were carried out. Then, samples were mounted and images were acquired using a Floid Cell Imaging Station (Life Technologies™).

**Figure S2.** (**A**) Changes in viability of ECs exposed to TGFβ1. ECs exposed to 0, 1, 2, 3, 4, 5 and 10 μg/ml TGFβ1 for 72 hrs, evaluated by means of (**A**) MTT assay and (**B**) propidium iodide (PI) incorporation assay. In (**A**), cell viability was expressed relative to the untreated (0 μg/ml TGFβ1) condition. In (**B**), cells incorporating PI (empty bars, PI^+^) denote cell death and PI-negative cells (filled bars, PI^+^) denote healthy cells. Statistical differences were assessed by a one-way anova (Kruskal–Wallis) followed by Dunn's *post hoc* test. **P* < 0.05 and ***P* < 0.01 against the untreated (0 μg/ml TGFβ1) condition. Graph bars show the mean ± S.D. (*N* = 3).

**Figure S3** Cellular distribution of proteins involved in TGFβ1-induced endothelial fibrosis. Representative images from the experiments of untreated (**A**–**H**) or 5 ng/ml TGFβ1-treated (**I**–**P**) ECs for 72 hrs. CD31 or VE-cadherin (red), and α-sma, FSP-1 or FN (green) were detected. The box depicted in **A**, **C**, **E** and **G** indicates the magnification shown in **B**, **D**, **F** and **H** respectively. Arrows indicate CD31 (**B** and **F**) or VE-Cadherin (**D** and **H**) labelling at the plasma membrane, whereas arrowheads indicate α-sma (**B**), FSP-1 (**D**) or FN (**F** and **H**) staining, indicating basal expression of fibrotic markers (**B** and **D**) or ECM proteins (**F** and **H**). The box depicted in **J**, **L**, **N** and **P** indicates the magnification shown in **I**, **K**, **M** and **O** respectively. Arrows indicate α-sma (**J**), FSP-1 (**L**) or FN (**N** and **P**) labelling in plasma membrane, whereas arrowheads indicate CD31 (**J** and **N**) or VE-Cadherin (**L** and **P**) staining from residual endothelial marker expression indicating EndMT. Nuclei were stained using DAPI. Bar scale represents 10 μm.

**Figure S4.** Unspecific staining in immunocytochemical experiments when primary antibodies were omitted. Experiments performed in the presence (+) or the absence (−) of primary antibodies against: CD31 (**A** and **B**), VE-cadherin (**C** and **D**), α-sma (**E** and **F**), fibronectin (**G** and **H**) and type III collagen (**I** and **J**). Arrows depicted unspecific staining. Nuclei were stained using DAPI. Bars scale represents 10 μm.

**Figure S5.** Integrity of the endothelial monolayer from intact whole blood vessels after 0 or 48 hrs of perfusion. (**A**–**D**) CD31 detection was performed in the endothelial monolayer from whole blood vessels perfused for 0 hr (**A**) or 48 hrs (**C**) in vehicle-perfused vessels. **B** and **D** show magnification of the box depicted in **A** and **C** respectively. Arrows indicate CD31 labelling at the plasma membrane. No significant changes were detected at 0 or 48 hrs of perfusion. Nuclei were stained using Hoechst. Bar scale represents 50 μm. (**E** and **F**) Structural integrity of endothelial monolayer from blood vessels was evaluated by haematoxylin eosin staining. Transversal slides from vehicle-perfused vessels incubated for 0 hr (**A**) or 48 hrs (**B**). Arrows indicate endothelial cells. Bar scale represents 50 μm.

**Figure S6.** Unspecific staining in immunohistochemical experiments when primary antibodies were omitted. Experiments performed in the presence (+) or absence (−) of primary antibodies against the following proteins: CD31 (**A** and **B**, in vehicle-perfused vessels), fibronectin (**C** and **D**, in LPS-perfused vessels) and type III collagen (**E** and **F**, in LPS-treated cells). Bar scale represents 50 μm.

**Figure S7.** ALK5 expression down-regulation by siRNA. Endothelial cells were transfected with a specific siRNA against human isoform of ALK5. (**A**). Representative images from western blot experiments performed for the detection of ALK5 in cells transfected with a siRNA against ALK5 (siALK5) or a non-targeting siRNA (siCTRL). (**B**) Densitometric analyses from several experiments, as shown in (**A**). Protein levels were normalized against tubulin, and the data are expressed relative to cells transfected with siCTRL condition. Statistical differences were assessed by student's *t*-test (Mann–Whitney). ****P* < 0.001. Graph bars show the mean ± S.D. (*N* = 3).
