# Supplementary material for: Lipopolysaccharide induces a fibrotic-like phenotype in endothelial cells
Source: J Cell Mol Med. 2013 May 2;17(6):800–14. doi: 10.1111/jcmm.12066 (PMC3823184; doi:10.1111/jcmm.12066)
Supplement: Supplementary file 1 [file jcmm0017-0800-SD1.pdf]

Supporting information

Lipopolysaccharide induces a fibrotic-like phenotype in endothelial cells.

Cesar Echeverría, Ignacio Montorfano, Daniela Sarmiento, Alvaro Becerra, Felipe Nuñez-Villena, Xavier Figueroa, Claudio Cabello-Verrugio, Alvaro Elorza, Claudia Riedel and Felipe Simon.

Detailed Methods References

Cells and vessels culture

Primary cells culture: Human umbilical vein endothelial cells (HUVEC) were isolated by collagenase (0.25 mg/mL) digestion from freshly obtained umbilical cord veins from normal pregnancies, after patient's informed consent. The Commission of Bioethics and Biosafety of Universidad Andres Bello approved all experimental protocols. The investigation also conforms with the principles outlined in the Declaration of Helsinki. Cells were grown in gelatin-coated dishes at 37°C in a 5%:95% CO_2:air atmosphere in medium 199 (Sigma, MO), containing 100 µg/mL endothelial cell growth supplement (ECGS) (Sigma), 100 µg/mL heparin, 5 mmol/L D-glucose, 3.2 mmol/L L-gutamine, 10% fetal bovine serum (FBS) (GIBCO, NY), and 50 U/mL penicillin-streptomycin (Sigma).

Blood vessels culture: intact 3-5 cm veins from human umbilical cord were cultured in HUVEC medium under the conditions described above. The interior of veins were perfused with medium containing the vehicle or LPS. Vein ends were clamped to avoid solution escape. A detailed protocol is described in *Figure S1*.

RNA isolation and quantitative real-time PCR

QPCR experiments were performed to measure CD31, VE-cadherin, α-sma, FSP-1, fibrinogen, and type III collagen mRNA levels in HUVEC cells. Total RNA was extracted with Trizol according to the manufacturer's protocol (Invitrogen, Carlsbad, CA). DNAse I-treated RNA was used for reverse transcription using the Super Script II Kit (Invitrogen, Carlsbad, CA). Equal amounts of RNA were used as templates in each reaction. QPCR was performed using the SYBR Green PCR Master Mix (AB Applied Biosystems, Foster City, CA). Assays were run using a Rotor-gene system (Corbet Research) instrument. Data are presented as relative mRNA levels of the gene of interest normalized to relative levels of 28S mRNA using the ΔCt method.[2] The following primers pairs were used: CD31, forward 5'-GAGAGTATTACTGCACAGCC-3′ and reverse 5′-GAGCAATGATCACTCCGATG-3′ (expected fragment size 140 bp); VE-cadherin, forward 5'-TGCATCCTCACCATCACAGT-3′ and reverse 5′-TTGAGCACCGACACATCGTA- 3′ (expected fragment size 172 bp); α-sma, forward 5'-CCAGCACCATGAAGATCAAG-3′ and reverse 5′- TTGCTGATCCACATCTGCTG- 3′ (expected fragment size 111 bp); FSP-1, forward 5'-GAGTACTGTGTCTTCCTGTC -3′ and reverse 5 ′-GCAGCTCCTTTAGTTCTGAC-3′ (expected fragment size 146 bp); Fibronectin, forward 5'-TCCATGATCTGGGACTGTAC-3′ and reverse 5′-CCAATCTTGTAGGACTGACC-3′ (expected fragment size 100 bp); Type III collagen, forward 5'-GCA TCA AAG GAC ATC GAG GA-3′ and reverse 5′-CAG AGG TGA AAG AGG ATC TG-3′ (expected fragment size 212 bp); 28S, forward 5'-CGA CGT CGC TTT TTG ATC CT-3′ and reverse 5′-ATG GCA AAC TGT CCA CAT GG-3′ (expected fragment size 245 bp).

Western blot procedures

Untreated or LPS-treated ECs were lysed in cold lysis buffer [150 mmol/L NaCl, 1 mmol/L EGTA, 50 mmol/L Tris, pH 7.4, 1% glycerol, 1% Triton X-100, 10 mmol/L NaF, 20 mmol/L NaPi, and protease inhibitor cocktail (Sigma)] and centrifuged (10,000×g for 15 min at 4°C), and then proteins were extracted. Supernatants were collected and stored in the same lysis buffer. Protein extract and supernatant were subjected to 12% (for FSP-1), 10% (for CD31, VE-cadherin, α-sma, p-smad2, total smad2, and tubulin), or 8% (for Col III and FN) SDS-PAGE. Resolved proteins were transferred to a nitrocellulose or PVDF membrane and non-specific binding was blocked using 5% BSA in PBS for 1 h at pH 7.4. The blocked membrane was incubated with the appropriate primary antibody, washed twice, and incubated with a secondary antibody. Bands were revealed using a peroxidase-conjugated IgG antibody. Tubulin was used as a loading control. For detection of the phosphorylated form of smad2 (p-smad2), we used an antibody against p-smad2 (p-ser 465/467) and total smad2 was used as a loading control. Peroxidase activity was detected through enhanced chemiluminescence (Bio-Rad, CA) and images were acquired using Fotodyne FOTO/Analyst Luminary Workstations Systems (Fotodyne, Inc., Hartland, WI). Protein content was determined by densitometric scanning of immunoreactive bands and intensity values were obtained by densitometry of individual bands normalized against tubulin or total smad. For a detailed list of antibodies used, see *Table S1*.

Fluorescent immunocytochemistry and immunohistochemistry

Immunocytochemistry: cells were washed twice with PBS and fixed with 3.7% PFA for 30 min at RT, treated with 50 mmol/L NH_4Cl for 15 min at RT, permeabilized with 0.1% Triton X-100 in PBS for 30 min at RT, and blocked for 2 h at RT with 3% BSA in PBS. The cells were

subsequently washed again and incubated with the first primary antibodies. Then, cells were washed twice and incubated with the first secondary antibodies. For immunofluorescent double staining, the cells were washed with PBS twice and the above staining procedure was repeated for the second set of primary and second secondary antibodies. Samples were mounted with ProLong Gold antifade mounting medium with DAPI (Invitrogen).

Immunohistochemistry: samples obtained from human umbilical cord vein were fixed using Bouin's fixative for 24 h at RT. Samples were permeabilized with 0.1% Triton X-100 in PBS for 40 min at RT and blocked for 3 h at RT with 3% bovine serum albumin in PBS. Samples were subsequently washed again and incubated with the first primary antibodies. Then, cells were washed twice and incubated with the first secondary antibodies. For immunofluorescent double staining, samples were washed with PBS twice and the above staining procedure was repeated for the second set of primary and second secondary antibodies. Nuclei were stained using Hoestch 33342 (Sigma). Images were acquired and analyzed using a Floid Cell Imaging Station (Life Technologies™). For a detailed list of antibodies used see *Table S2*.

Immunohistochemistry by hematoxylin-eosin stain

Human umbilical cord vein were fixed in 10% buffer formalin solution. The veins were sectioned using a cryostat (Leica), and slices (15 μm) were prepared with hematoxylin-eosin stain and examined with light microscopy.

Cell viability determination

MTT assay: cell viability was evaluated using the 3-(4,5-dimethylthiazol-2-yl)-2,5-diphenyltetrazolium bromide (MTT) colorimetric assay (Invitrogen, Eugene, Oregon, USA), in which cell viability was quantified by the amount of MTT reduction.[1] After different treatments were performed, cells were co-incubated with anhydrous MTT (0.5 mg/mL) for 4 h and then solubilized with an isopropanol/DMSO solution. The optical density value was measured at 540 nm. At least three separate experiments were performed in triplicate. The data are expressed as cell viability normalized against untreated cells.

Propidium iodide incorporation assay: After treatments, total HUVEC cells were harvested by centrifugation at 800 x g for 5 min, and the pellet was suspended in 200 µl PBS. Then, cells were washed once with PBS and stained with propidium iodide (PI, 10 µg/mL) for 20 min at room temperature in the dark. DNA content was analyzed with a flow cytometry system (FACSCanto, BD Biosciences, CA, USA). A minimum of 10,000 cells/sample was analyzed. PI intensity analysis was performed using FACSDiva software (BD Biosciences, CA, USA).

Small interfering RNA against ALK5 and transfection

SiGENOME SMARTpool siRNA (four separated siRNAs per human ALK5 transcript) were purchased from Dharmacon (Dharmacon, Lafayette, CO). The following siRNA were used: human ALK5 (siRNA-ALK5) and non-targeting siRNA (siRNA-CTRL) used as a control. In brief, HUVEC were plated overnight in 24-well plate and then transfected with 5 nmol/L siRNA using DharmaFECT 4 transfection reagent (Dharmacon) used according to the manufacturer's protocols in serum-free medium for 6 hours. After 24 to 48 transfection, experiments were performed.

Reagents

Lipopolysaccharide from *E. coli* was purchased from Sigma (0127:B8). ALK5 inhibitor, SB431542; specific smad3 inhibitor, SIS3; were purchased from Tocris. Apocynin and NAC was purchased from Sigma-Aldrich. TLR4 inhibitor, CLI-095 was purchased from InvivoGen. TGFβ1 and TGFβ2 were purchased from R&D Systems. Buffers and salts were purchased from Merck Biosciences (Darmstadt).

Data analysis

All results are presented as the mean±SD. An ANOVA followed by the Bonferroni or Dunn's *post hoc* tests were used and considered significant at $p<0.05$.

Detailed Methods References

1. Mosmann T. Rapid colorimetric assay for cellular growth and survival: application to proliferation and cytotoxicity assays. *J Immunol Methods* 1983;**65**:55-63.
2. Pfaffl MW. A new mathematical model for relative quantification in real-time RT-PCR. *Nucleic Acids Res* 2001;**29**:2002-2007.

Table S1. Primary and secondary antibodies used in western blot experiments

Primary Ab	*Dilution*	*Source*	*Incubation time*	*Incubation temperature*	*Brand*
CD31	1:1000	Mouse	2 h	RT	Dako, Denmark
VE-cadherin	1:500	Goat	2 h	RT	Santa Cruz, CA, USA
αSMA	1:1000	Rabbit	2 h	RT	Millipore, MA, USA
FSP-1	1:500	Mouse	2 h	RT	Abcam, MA, USA
Fibronectin	1:6000	Rabbit	2 h	RT	Sigma-Aldrich, MO, USA
Type III collagen	1:6000	Rabbit	2 h	RT	Rockland, PA, USA
p-smad2	1:1000	Rabbit	2 h	RT	Calbiochem, SA, USA
smad2	1:1000	Mouse	2 h	RT	Cell Signaling, MA, USA

Secondary Ab	*Dilution*	*Source*	*Incubation time*	*Incubation temperature*	*Brand*
Anti- rabbit HRP	1:5000	Goat	2 h	RT	Millipore, MA, USA
Anti- mouse HRP	1:5000	Goat	2 h	RT	Millipore, MA, USA
Anti- Goat HRP	1:6000	Rabbit	2 h	RT	Abcam, MA, USA

Ab: antibody, RT: room temperature, ON: over night

Table S2. Primary and secondary antibodies used in immunocytochemistry and immunohistochemistry.

Immunocytochemistry

Primary Ab	*Dilution*	*Source*	*Incubation time*	*Incubation temperature*	*Brand*
CD31	1:200	Mouse	ON	4ºC	Dako, Denmark
VE-cadherin	1:100	Goat	ON	4ºC	Santa Cruz, CA, USA
αSMA	1:500	Rabbit	ON	4ºC	Millipore, MA, USA
FSP-1	1:100	Mouse	ON	4ºC	Abcam, MA, USA
Fibronectin	1:400	Rabbit	ON	4ºC	Sigma-Aldrich, MO, USA
Type III collagen	1:200	Rabbit	ON	4ºC	Rockland, PA, USA

Secondary Ab	*Dilution*	*Source*	*Incubation time*	*Incubation temperature*	*Brand*
Anti- rabbit Alexa-488	1:250	Goat	2 h	RT	Invitrogen, CA, USA
Anti- goat Alexa-594	1:250	Donkey	2 h	RT	Invitrogen, CA, USA
Anti- mouse Alexa-488	1:250	Goat	2 h	RT	Invitrogen, CA, USA
Anti- mouse Alexa-594	1:250	Goat	2 h	RT	Invitrogen, CA, USA

Immunohistochemistry

Primary Ab	*Dilution*	*Source*	*Incubation time*	*Incubation temperature*	*Brand*
CD31	1:100	Mouse	ON	4°C	Dako, CA, USA
Fibronectin	1:200	Rabbit	ON	4°C	Sigma-Aldrich, MO,USA
Type III collagen	1:100	Rabbit	ON	4°C	Rockland, MA,USA

Secondary Ab	*Dilution*	*Source*	*Incubation time*	*Incubation temperature*	*Brand*
Anti-rabbit Alexa-488	1:250	Goat	2h	RT	Invitrogen, CA, USA
Anti- mouse Alexa-594	1:250	Goat	2h	RT	Invitrogen, CA, USA

Ab: antibody, RT: room temperature, ON: over night

Supplementary Figure Legends

Figure S1 Culturing of intact whole blood vessel with differential perfusion. (**A**) Veins from human umbilical cord were isolated and incubated for 48 h. External solution contained isotonic medium containing FBS and growth factors. Internal solution was HUVEC medium containing vehicle or 20 µg/mL LPS. External and internal solution were not mixed during experiments. Solutions were changed frequently. (**B**) Cultured blood vessel was dissected to extract a small portion of vessel wall to exposed ECs monolayers. Samples extracted were 150 mm^2 approximately. Vessel wall structure was not altered. (**C**) Monolayer samples were placed in a coverslip and immunohistochemistry experiments were carried out. Then, samples were mounted and images were acquired using a Floid Cell Imaging Station (Life Technologies™).

Figure S2. (A). Changes in viability of ECs exposed to TGFβ1. ECs exposed to 0, 1, 2, 3, 4, 5 and 10 µg/mL TGFβ1 for 72 h, evaluated by means of (A) MTT assay and (B) propidium iodide (PI) incorporation assay. In (A), cell viability was expressed relative to the untreated (0 µg/mL TGFβ1) condition. In (B), cells incorporating PI (empty bars, PI^+) denote cell death and PI negative cells (filled bars, PI^+) denote healthy cells. Statistical differences were assessed by a one-way analysis of variance (ANOVA) (Kruskal–Wallis) followed by Dunn's post hoc test. *: $P < 0.05$ and **: P < 0.01 against the untreated (0 µg/mL TGFβ1) condition. Graph bars show the mean ± SD ($N = 3$).

Figure S3 Cellular distribution of proteins involved in TGFβ1-induced endothelial fibrosis. Representative images from experiments of untreated (**A**–**H**) or 5 ng/mL TGFβ1-treated (**I**–**P**) ECs for 72 h. CD31 or VE-cadherin (red), and α-sma, FSP-1, or FN (green) were detected.

The box depicted in (**A**, **C**, **E**, and **G**) indicates the magnification shown in (**B**, **D**, **F**, and **H**), respectively. Arrows indicate CD31 (**B**, **F**) or VE-Cadherin (**D**, **H**) labeling at the plasma membrane, whereas arrowheads indicate α-sma (**B**), FSP-1 (**D**), or FN (**F**, **H**) staining, indicating basal expression of fibrotic markers (**B**, **D**) or ECM proteins (**F**, **H**). The box depicted in (**J**, **L**, **N**, and **P**) indicates the magnification shown in (**I**, **K**, **M**, and **O**), respectively. Arrows indicate α-sma (**J**), FSP-1 (**L**), or FN (**N**, **P**) labeling in plasma membrane, whereas arrowheads indicate CD31 (**J**, **N**) or VE-Cadherin (**L**, **P**) staining from residual endothelial marker expression indicating EndMT. Nuclei were stained using DAPI. Bar scale represents 10 μm.

Figure S4. Unspecific staining in immunocytochemical experiments when primary antibodies were omitted. Experiments performed in the presence (+) or the absence (-) of primary antibodies against: CD31 (**A**–**B**), VE-cadherin (**C**–**D**), α-sma (**E**–**F**), fibronectin (**G**–**H**), and type III collagen (**I**–**J**). Arrows depicted unspecific staining. Nuclei were stained using DAPI. Bars scale represents 10 μm.

Figure S5. Integrity of the endothelial monolayer from intact whole blood vessels after 0 or 48 h of perfusion. (**A-D**) CD31 detection was performed in the endothelial monolayer from whole blood vessels perfused for 0 h (**A**) or 48 h (**C**) in vehicle-perfused vessels. (**B** and **D**) show magnification of the box depicted in (**A** and **C**), respectively. Arrows indicate CD31 labeling at the plasma membrane. No significant changes were detected at 0 or 48 h of perfusion. Nuclei were stained using Hoechst. Bar scale represents 50 μm. (**E-F**) Structural integrity of endothelial monolayer from blood vessels was evaluated by hematoxylin eosin staining. Transversal slides from vehicle-perfused vessels incubated for 0 h (**A**) or 48 h (**B**). Arrows indicate endothelial cells. Bar scale represents 50 μm.

Figure S6. Unspecific staining in immunohistochemical experiments when primary antibodies were omitted. Experiments performed in the presence (+) or absence (-) of primary antibodies against to the following proteins: CD31 (**A**–**B**, in vehicle-perfused vessels), fibronectin (**C**–**D**, in LPS-perfused vessels), and type III collagen (**E**–**F**, in LPS-treated cells). Bar scale represents 50 µm.

Figure S7. ALK5 expression downregulation by siRNA. Endothelial cells were transfected with a specific siRNA against human isoform of ALK5. (**A**). Representative images from western blot experiments performed for detection of ALK5 in cells transfected with a siRNA against ALK5 (siALK5) or a non-targeting siRNA (siCTRL). (**B**). densitometric analyses from several experiments, as shown in (**A**). Protein levels were normalized against tubulin, and the data are expressed relative to cells transfected with siCTRL condition. Statistical differences were assessed by student's t-test (Mann-Whitney). ***: $P < 0.001$. Graph bars show the mean ± SD ($N = 3$).

Figure S1

A **FASE I:**
INTACT BLOOD VESSEL INCUBATION

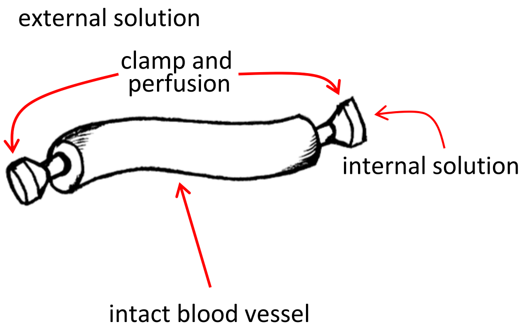

B **FASE II:**
DISSECTION TO ECs MONOLAYER EXPOSITION

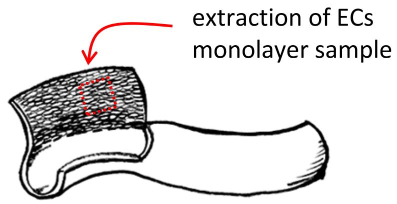

C **FASE III:**
INMUNOHISTOCHEMISTRY AND MICROSCOPY

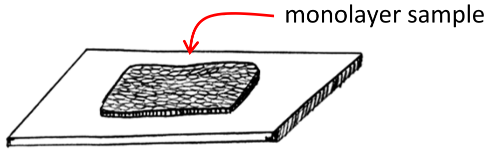

Figure S2

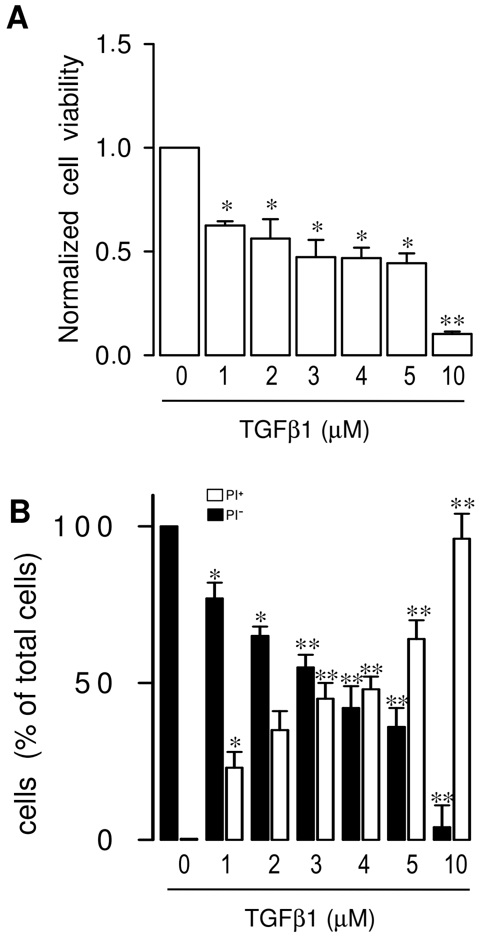
A
Normalized cell viability
1.5
1.0
0.5
0.0
*
*
*
*
*
**
0 1 2 3 4 5 10
TGFβ1 (μM)
B
PI+
PI−
cells (% of total cells)
100
50
0
0 1 2 3 4 5 10
TGFβ1 (μM)

Figure S3

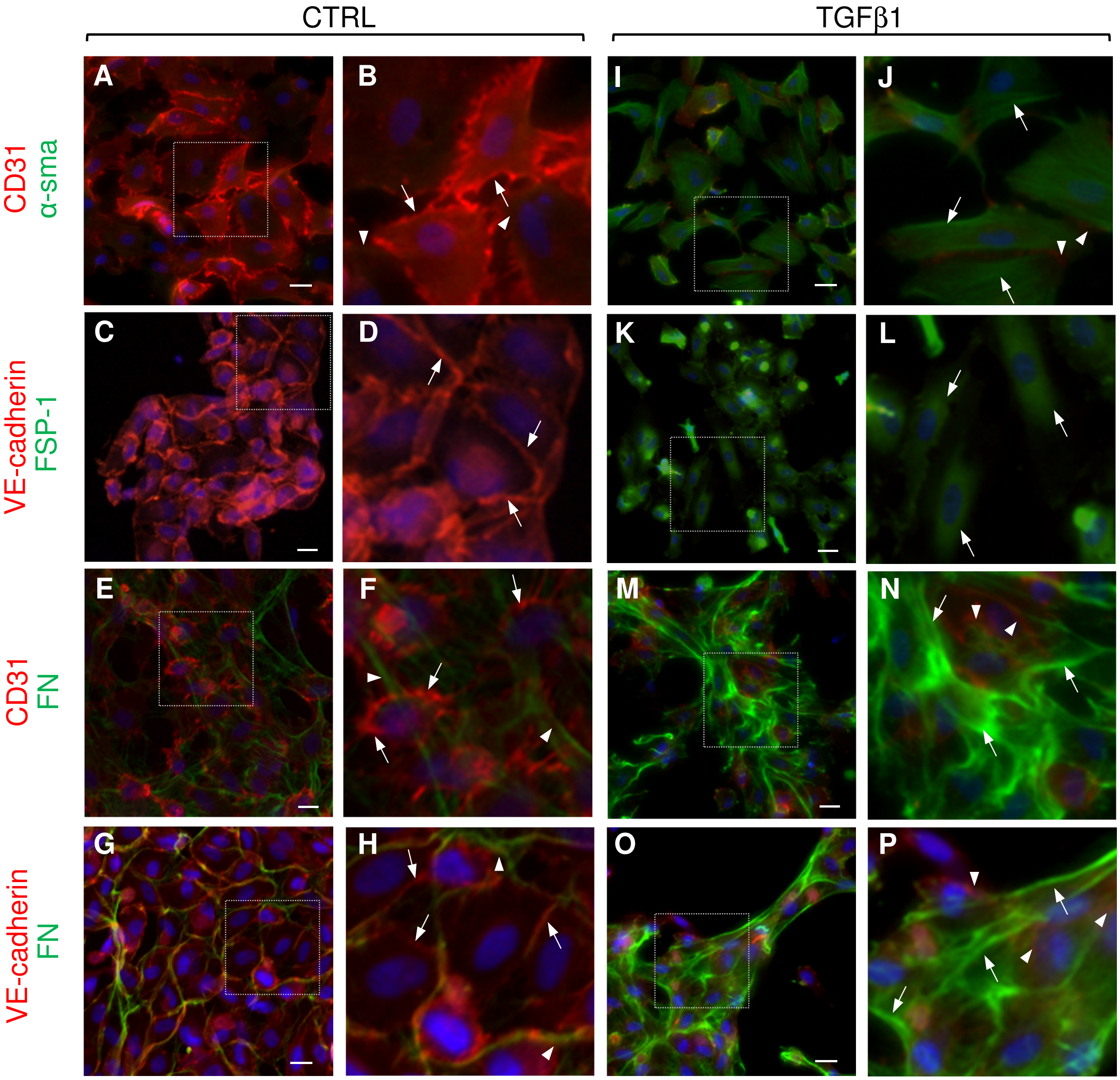
CTRL
TGFβ1
CD31
α-sma
VE-cadherin
FSP-1
CD31
FN
VE-cadherin
FN
A
B
C
D
E
F
G
H
I
J
K
L
M
N
O
P

Figure S4

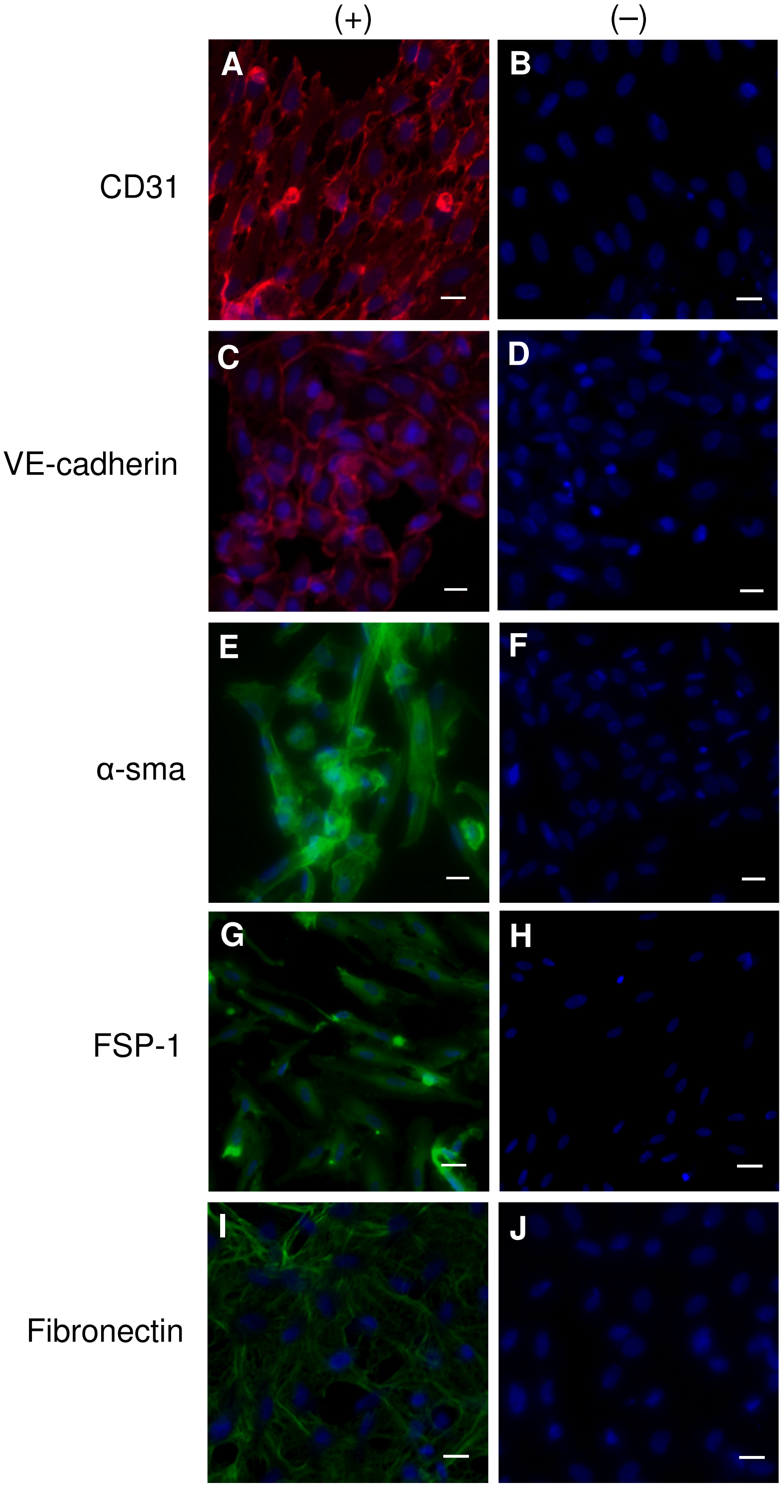

Figure S5

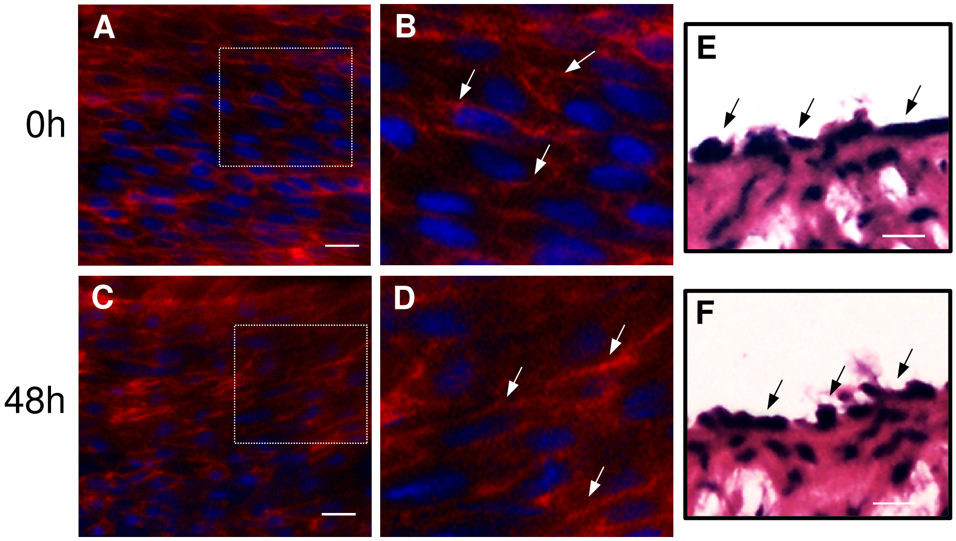

Figure S6

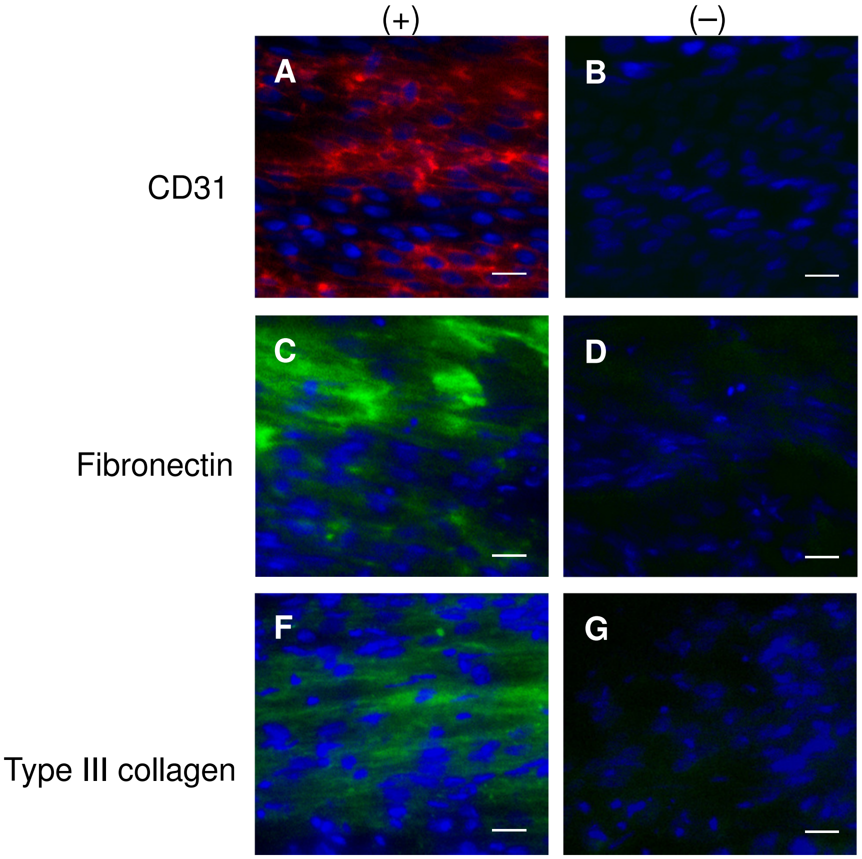

Figure S7

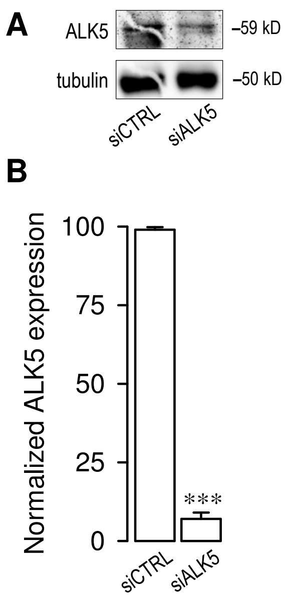
A
ALK5
–59 kD
tubulin
–50 kD
siCTRL
siALK5
B
Normalized ALK5 expression
100
75
50
25
0
***
siCTRL
siALK5
